# Determinants of astrocytic pathology in stem cell models of primary tauopathies

**DOI:** 10.1186/s40478-023-01655-1

**Published:** 2023-10-06

**Authors:** Kimberly L. Fiock, Jordan N. Hook, Marco M. Hefti

**Affiliations:** 1 Department of Pathology, University of Iowa, 25 S Grand Ave MRC-108-A, Iowa City, IA 52240 USA; 2 Experimental Pathology Graduate Program, University of Iowa, Iowa City, IA USA; 3 Carver College of Medicine, University of Iowa, Iowa City, IA USA; 4 Iowa Neuroscience Institute, University of Iowa, Iowa City, IA USA

**Keywords:** Astrocytes, Stem cells, Tauopathy, Tau, Progressive supranuclear palsy, Corticobasal degeneration

## Abstract

Astrocytic tau aggregates are seen in several primary and secondary tauopathies, including progressive supranuclear palsy (PSP), corticobasal degeneration (CBD), and chronic traumatic encephalopathy (CTE). In all of these diseases, astrocytic tau consists mostly of the longer (4R) tau isoform, even when adjacent neuronal aggregates consist of a mixture of 3- and 4R tau, as in CTE. Even the rare astrocytic tau aggregates seen in Pick’s disease appear to contain both 3R and 4R tau. The reasons for this, and the mechanisms by which astrocytic tau aggregates form, remain unclear. We used a combination of RNA in situ hybridization and immunofluorescence in post-mortem human brain tissue, as well as tau uptake studies in human stem cell-derived astrocytes, to determine the origins of astrocytic tau in 4R tauopathies. We found no differences in tau mRNA expression between diseases or between tau positive and negative astrocytes within PSP. We then found that stem cell-derived astrocytes preferentially take up long isoform (4R) recombinant tau and that this uptake is impaired by induction of reactivity with inflammatory stimuli or nutritional stress. Astrocytes exposed to either 3R or 4R tau also showed downregulation of genes related to astrocyte differentiation. Our findings suggest that astrocytes preferentially take up neuronal 4R tau from the extracellular space, potentially explaining why 4R tau is the predominant isoform in astrocytic tau aggregates.

**Supplementary Information:**

The online version contains supplementary material available at 10.1186/s40478-023-01655-1.

## Introduction

**Fig. 1:**
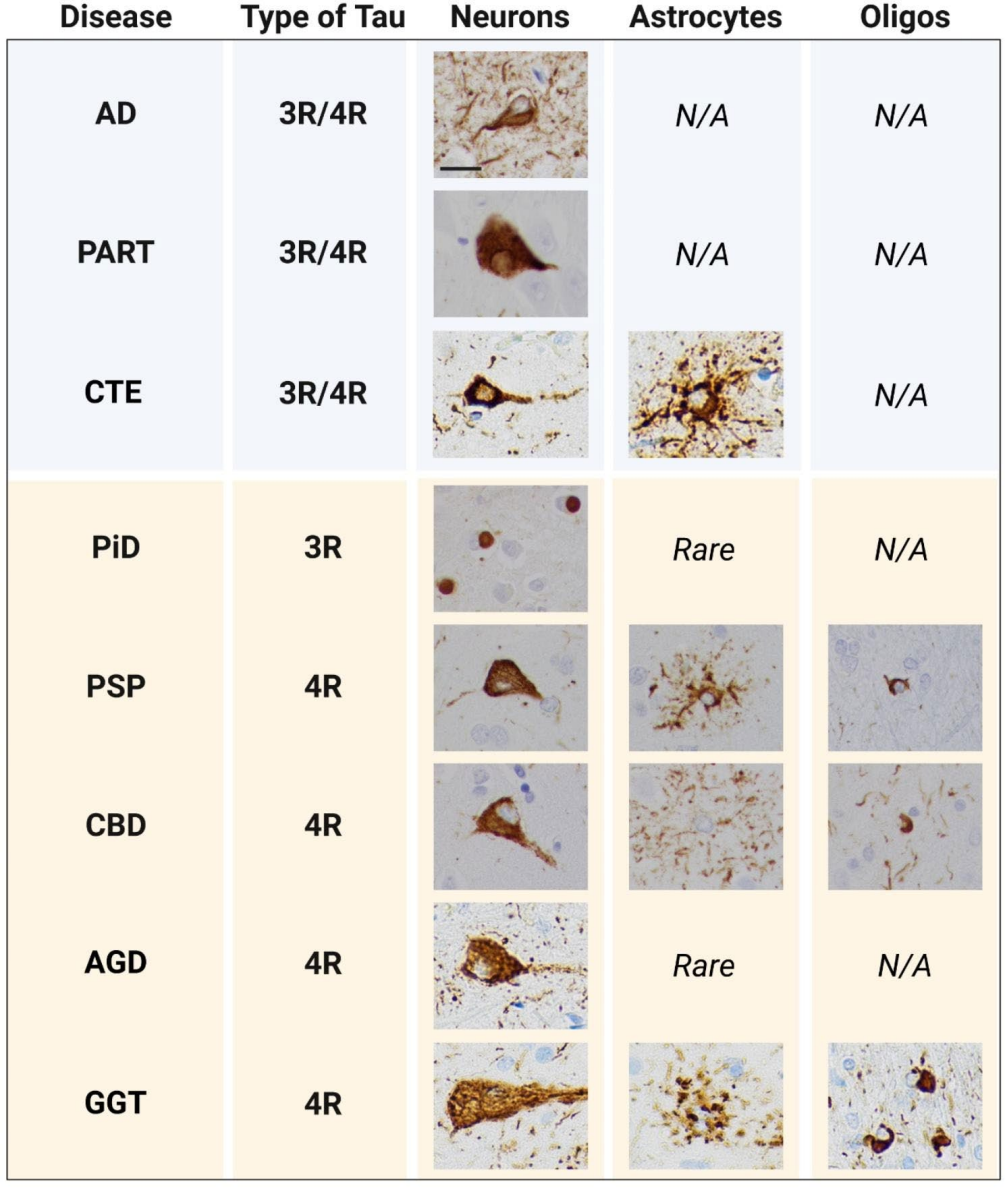
Tau pathology across diseases. Tau pathology is found exclusively in neurons in AD and PART, which are mixed 3R/4R tauopathies. In 4R tauopathies, such as PSP, CBD, and GGT, tau pathology is seen in neurons, astrocytes, and oligodendrocytes. Both PiD, a 3R tauopathy, and AGD, a 4R tauopathy, show primarily neuronal pathology with rare astrocytic involvement. Although neuronal inclusions in CTE are composed of both 3R and 4R tau, the astrocytic pathology is predominantly 4R. Scale bar = 20 μm

Neurodegenerative tauopathies can be classified based on the composition of pathological tau aggregates into short (3R) isoform-predominant, long (4R) isoform-predominant, or mixed (3R/4R). Astrocytic tau is characteristic of 4R tauopathies such as corticobasal degeneration (CBD) or progressive supranuclear palsy (PSP), where its isoform composition reflects that of adjacent neuronal tau aggregates. However, even when it is seen in mixed tauopathies such as chronic traumatic encephalopathy (CTE), it is composed predominantly of 4R tau [4] (Fig. 1).

The classic neuropathologic findings in CBD and PSP are astrocytic plaques and tufted astrocytes, respectively. Astrocytic plaques are composed of tau aggregates in distal astrocytic processes, while tufted astrocytes show tau aggregation in proximal astrocytic processes and the soma (Fig. 1). The 4R tauopathies globular glial tauopathy (GGT) and argyrophilic grain disease (AGD) also both show astrocytic pathology, although it is rare in AGD. By contrast, tau pathology in Pick’s disease (PiD) (3R), Alzheimer’s disease (AD) (3R/4R), and primary age-related tauopathy (PART) (3R/4R) is largely limited to neurons, although rare astrocytic pathology with variable mixtures of 3R and 4R tau has been reported in Pick’s disease [2, 9, 10, 19, 20] (Fig. 1). Even in CTE, where the pathognomonic neuronal tau aggregates in the depths of sulci are composed of a mixture of 3R and 4R tau, astrocytic tau aggregates consist almost entirely of 4R [4] (Fig. 1). The reasons for this predominance of 4R tau pathology in astrocytes remain unclear.

In rodent models, astrocytic tau cannot propagate in the absence of neuronal tau expression [26], and human single-cell sequencing and RNA in situ hybridization data do not show an upregulation of tau expression in astrocytes from PSP patients [13, 27]. Astrocytes will also readily take up recombinant tau fibrils or monomers in vitro [14, 17, 33]. It is therefore likely that astrocytic tau is primarily of neuronal origin. The human data are however based on a small number of cases, and CBD has not been systematically studied. The ability of astrocytes to take up different tau isoforms (e.g., 3R vs. 4R), and the downstream effects of such uptake also remain unclear. There has been considerable debate as to whether astrocytic tau pathology represents a degenerative or a reactive process, and if the latter, what the underlying cellular mechanisms are [8, 16, 18, 22–24, 32, 34].

Here, we report the first systematic assessment of astrocytic tau expression in human brain tissue across multiple neurodegenerative disorders and the novel finding that astrocytes preferentially take up 4R tau monomers, which is impaired by exposure to inflammatory stimuli or nutritional stress.

## Results

### Astrocytic tau in human 4R tauopathies is of neuronal origin

**Fig. 2:**
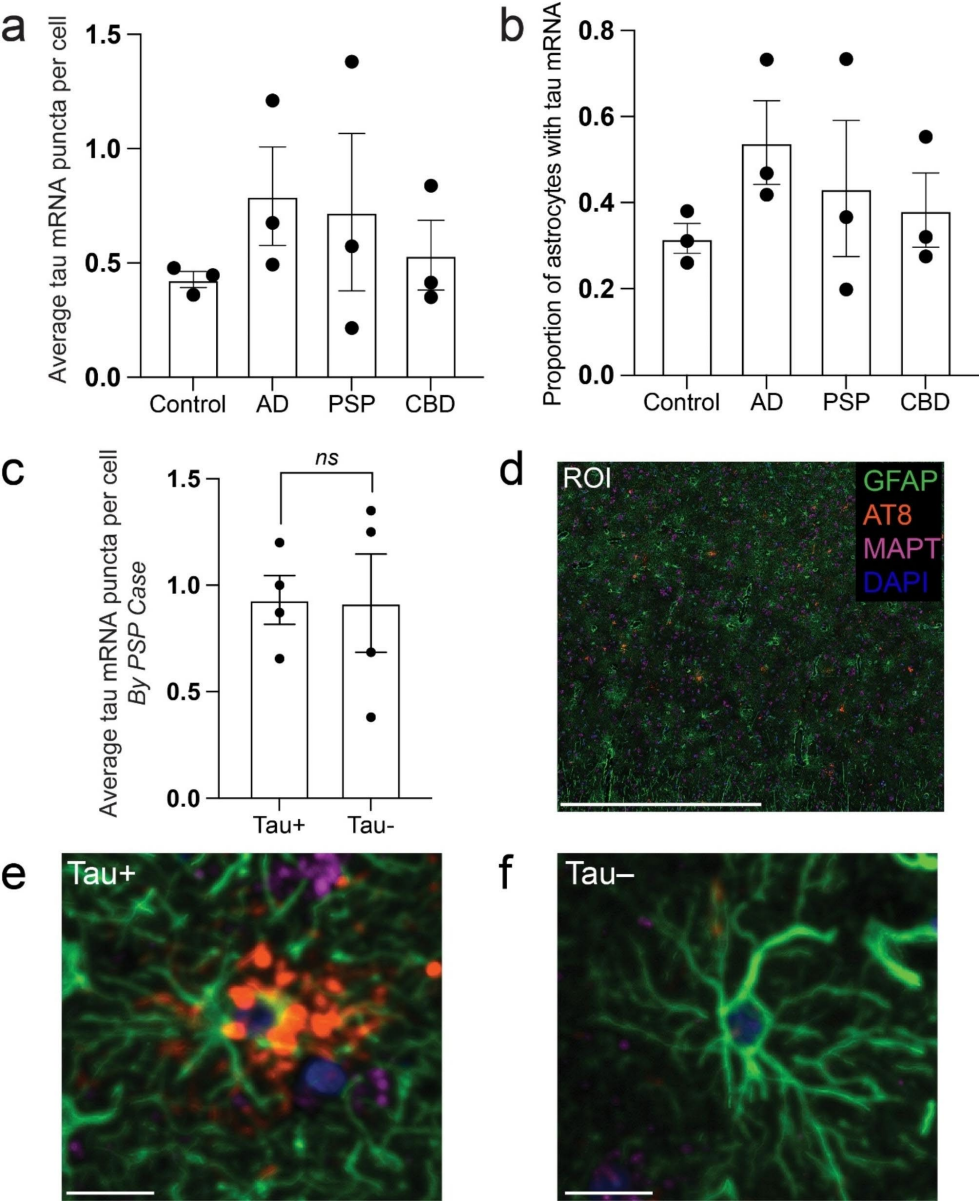
Astrocytes do not upregulate tau mRNA in astrocytic tauopathies. (**A**) Mean number of mRNA puncta per astrocyte in control, AD, PSP, and CBD. (**B**) Proportion of tau mRNA positive astrocytes in control, AD, PSP, and CBD. (**C**) Mean number of puncta comparing tau-positive and negative astrocytes within cases of PSP. (**D**) Representative low power ROI with insets of tau-positive and negative astrocytes shown in (**E**) and (**F**), respectively. Scale bars = 1000 μm in (**D**) and 20 μm in (**E**) and (**F**); n = 3 individual cases per condition in (**A**) and (**B**) and n = 4 individual cases in (**C**)

We first sought to determine whether astrocytes in PSP and CBD, both characterized by astrocytic tau aggregates, increase their expression of tau mRNA. Building upon our existing data using combined RNA in situ hybridization (RNAscope) and immunofluorescence, we found that the mean number of *MAPT* (tau) mRNA puncta per astrocyte (defined by positive immunofluorescence for GFAP) (Fig. 2a) and the proportion of astrocytes with puncta (Fig. 2b) did not differ significantly between adult control, AD, PSP, and CBD cases [11]. *MAPT* (tau) mRNA puncta were rare in astrocytes in all conditions, occurred as single dots, and were seen in both the nucleus and cytoplasm. The number of astrocytes per case was 3,204 ± 401 with no significant difference between groups by one-way ANOVA (p = 0.3571). We then sought to determine whether astrocytes containing tau aggregates (GFAP^+^AT8^+^) had higher levels of tau mRNA than those without (GFAP^+^AT8^−^). Because the pattern of tau staining in PSP astrocytes allows ready identification of the astrocytic soma, unlike more diffuse astrocytic plaques, we focused this analysis on cases of PSP. Comparing GFAP^+^AT8^+^ to GFAP^+^AT8^−^ astrocytes *within* cases of PSP, we found no statistically significant difference in the mean number of puncta per astrocyte (Fig. 2c) or in the number of astrocytes expressing tau mRNA (not shown). The mean number of astrocytes per case was 3,284 ± 392.

### hESC-derived astrocytes preferentially take up 4R tau

**Fig. 3:**
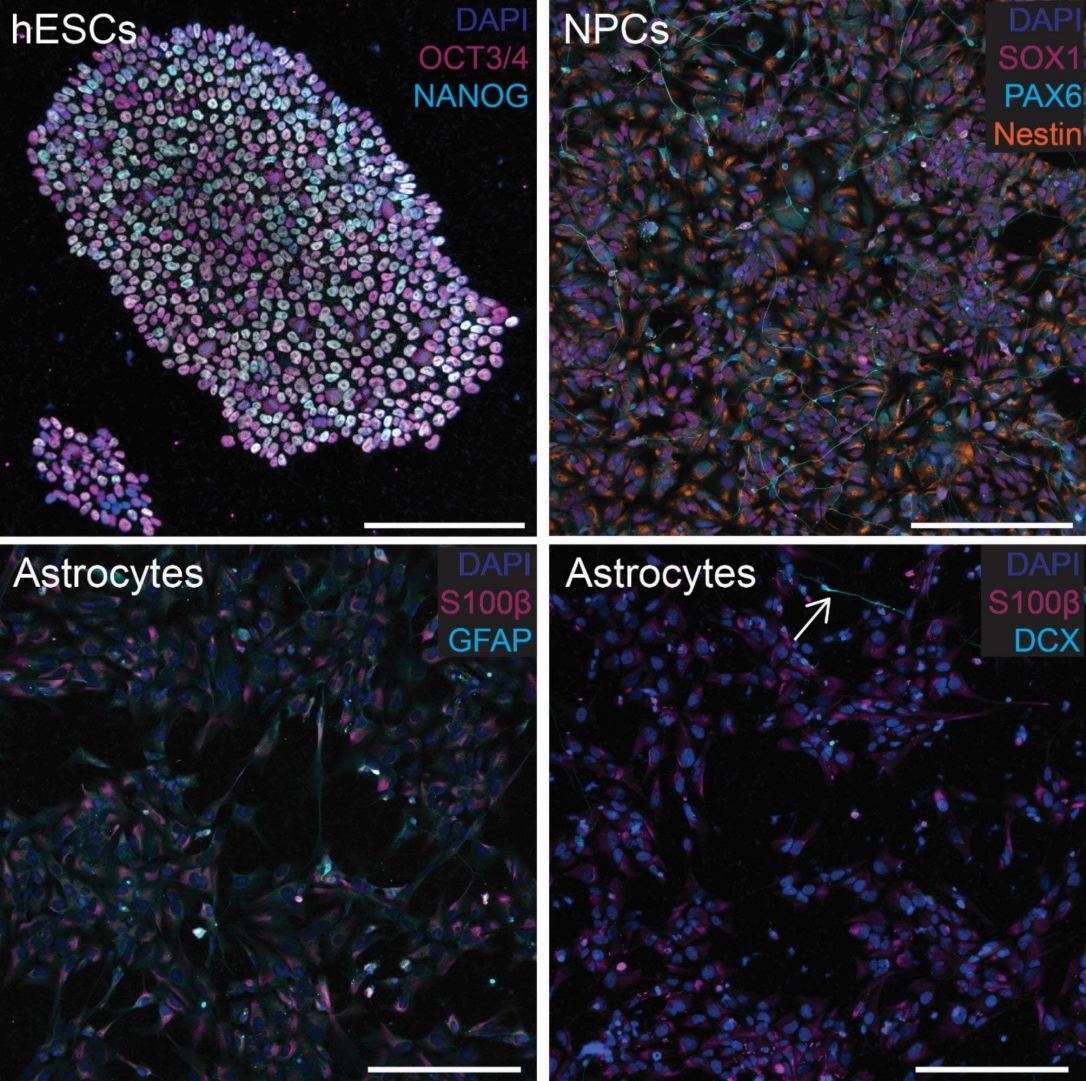
Validation of cell differentiation. Stem cells show positive staining for OCT3/4 and NANOG (top left), and neuronal precursor cells stain positive for Nestin, SOX1, and PAX6 (top right). Astrocytes show positive staining for S100β and GFAP (bottom left) with little to no DCX (bottom right). A single cell showing neuronal differentiation is indicated by white arrow (bottom right). Scale bars = 200 μm. Individuals panels for each stain are shown in Figs. S1, S2, and S3 for stem cells, neural progenitors, and astrocytes, respectively

**Fig. 4:**
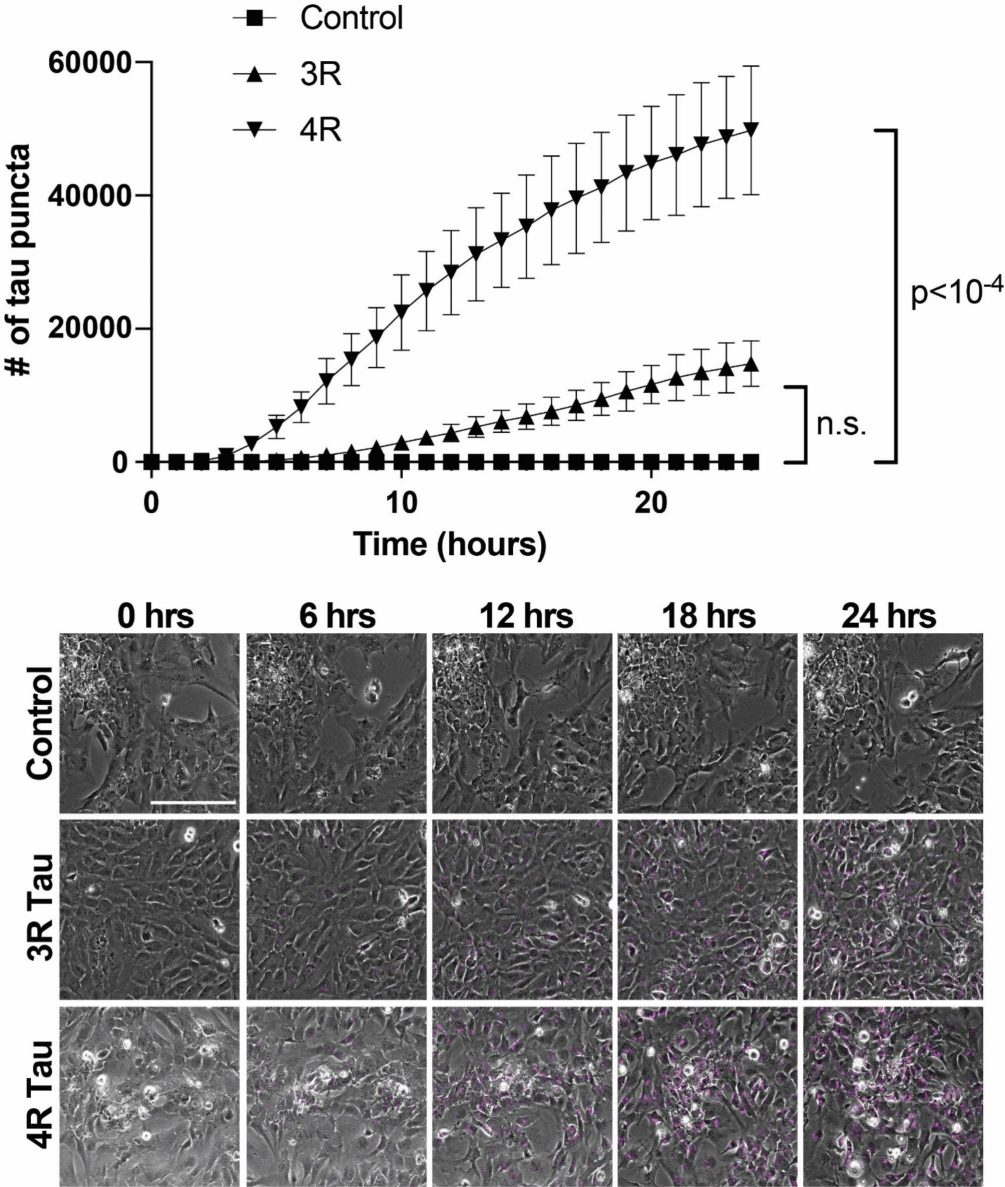
hESC-derived astrocytes preferentially take up 4R tau. hESC-derived astrocytes exposed to either labeled recombinant 3R or 4R tau over the course of 24 h exhibit preferential uptake of 4R. Image stills of hESC-derived astrocytes exposed to labeled recombinant 3R or 4R tau (magenta) over the course of 24 h show more 4R in cells than 3R. Scale bar = 200 μm; n = 3 biological replicates per condition

Next, we wanted to characterize the ability of human stem cell-derived astrocytes to take up 3R and 4R tau using labeled recombinant (monomeric) tau. Stem cells were validated by immunocytochemistry and trilineage differentiation, and all subsequent differentiation steps were validated by immunocytochemistry (Fig. 3, and S1-S4). Recombinant tau produced in *E. coli* was validated by SDS-PAGE, followed by Coomassie Blue staining and western blotting (Fig. S5a and S5b, respectively). When cultured with Cy5-labeled recombinant 1N3R or 1N4R tau, astrocytes took up significantly more 4R than 3R tau (p < 10^− 4^), with 4R uptake also occurring earlier than 3R (Fig. 4). This result was further validated with a separate, independently differentiated, batch of astrocytes (Fig. S6).

### Astrocytic uptake of tau impairs astrocyte maturation

**Fig. 5:**
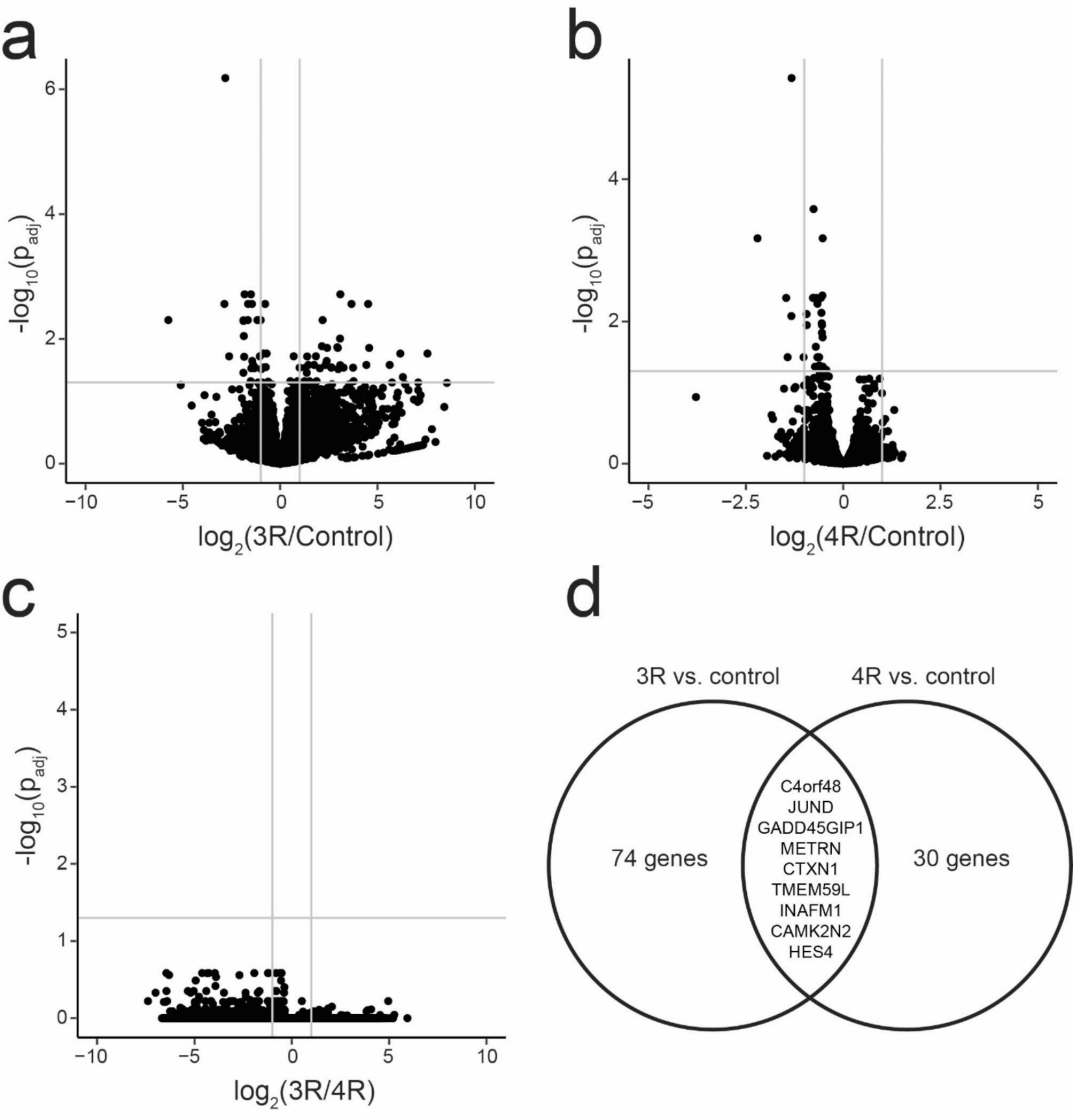
Effect of 3R and 4R tau uptake on astrocyte transcriptome. Volcano plots showing differentially expressed genes between 3R and control (**A**), 4R and control (**B**), and 3R and 4R (**C**). Genes differentially expressed in both 3R and 4R are shown in (**D**). Grey lines in (**A**) – (**C**) indicate cutoffs of adjusted p-value > 0.05 (horizontal) and absolute fold change >  2 (vertical)

We then used bulk RNA sequencing to compare the transcriptomes of 3R and 4R-treated astrocytes to untreated controls. We found 74 genes that differed between 3R-treated and control astrocytes, 30 between 4R-treated and control, and no differentially expressed genes between 3R- and 4R-treated astrocytes. Nine genes were significantly downregulated in both the 3R- and 4R-exposed conditions compared to controls (Fig. 5). For downstream analysis, we focused on genes that showed at least a two-fold difference in each comparison (60 between 3R-treated and control astrocytes and 6 between 4R-treated and control astrocytes). Of the 60 genes with at least two-fold differential expression in the 3R vs. control comparison, 36 were upregulated and 24 downregulated. The 36 genes with two-fold upregulation in the 3R condition showed a protein-protein interaction (PPI) enrichment p-value of 0.00114, indicating more interactions than expected by chance, and enrichment for GOCC:0070062 (FDR = 0.0468). The 24 two-fold downregulated genes in the 3R condition showed a PPI enrichment p-value of 0.0348 and no significant gene ontology enrichment. The six genes with two-fold downregulation in 4R tau were too few for meaningful enrichment analysis. Five genes showed significant downregulation by at least a factor of two in both comparisons (*C4orf48, HES4, INAFM1, TMEM59L, CAMK2N2*). Of these down-regulated genes, *C4orf48*, *HES4, INAFM1*, and *TMEM59L* promote stem cell or astrocyte differentiation, which suggests that tau uptake impairs astrocyte differentiation [1, 7, 12].

### Astrocytes exposed to inflammatory stimuli or nutritional stress have impaired tau uptake and degradation

**Fig. 6:**
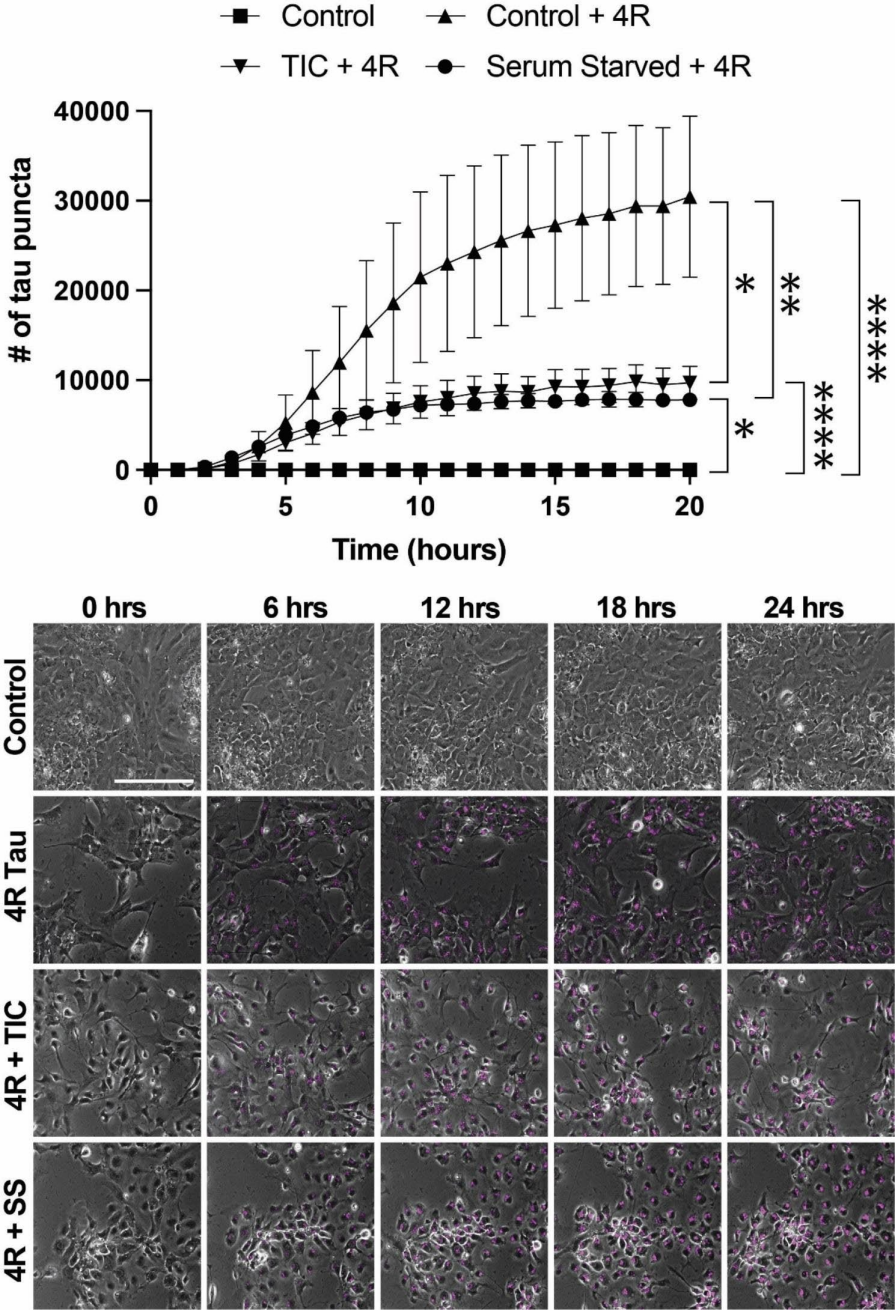
Nutritional or inflammatory stress impairs astrocyte tau uptake. hESC-derived astrocytes exposed to labeled 4R tau with or without serum starvation (SS) or TIC protocol (TNFα, IL1-α, C1q) with representative still images. N = 3 technical replicates per condition; *p = 0.0015, **p = 0.0005, ***p = 0.0001, ****p < 10 − 5

We then sought to test the effect of two distinct models of astrocytic stress on astrocytic tau uptake. We chose to focus on serum starvation, a model of nutritional stress, and TNF-alpha, IL1-alpha, and C1q (“TIC”), a model of inflammatory reactive astrocytes [24]. The two models showed identical results, with both taking up significantly less 4R tau than their control counterparts (TIC vs. control, p = 0.0015 and serum starved vs. control, p = 0.0005) (Fig. 6). Interestingly, both conditions also reached a plateau in the amount of 4R taken up after roughly 10 h, compared to the control condition that saw continuous uptake over the 24-hour period (Fig. 6).

## Discussion

Our data shows that astrocytes do not increase tau expression in progressive supranuclear palsy or corticobasal degeneration. This is true *between* cases, when PSP or CBD are compared to controls, and *within* cases, comparing tau-positive and tau-negative astrocytes in patients with PSP. It should be noted however, that our data does not allow us to exclude the possibility of a difference between tau-containing and tau-negative astrocytes in CBD. We also showed that stem cell-derived astrocytes preferentially take up 4R rather than 3R tau, that this downregulates the expression of genes related to astrocyte differentiation, and that is impaired by nutritional or inflammatory stress. Our finding that astrocytes take up 4R tau in greater quantity and more rapidly than 3R suggests a possible mechanism for the 4R tau aggregates seen in astrocytic tauopathies such as PSP and CBD. The fact that both inflammatory and nutritional stresses cause a plateau in the amount of 4R tau taken up after 10 h suggests a possible impairment of degradation in the stressed astrocytes, where they cannot take up more tau due to a lack of clearance of the tau they already have. This is an unexpected result as, given the homeostatic role of astrocytes, we would have anticipated *increased* uptake of tau in response to stress, and will require further studies to identify the underlying mechanisms.

Our findings are broadly in agreement with single nuclear sequencing and RNA in situ hybridization studies in PSP [13, 27]. Our work is also complementary to and builds on data presented by Forrest et al. in that we use GFAP immunofluorescence to identify astrocytic mRNAs and include a larger number of cases [13]. We also examined astrocytic tau expression in AD and CBD, which have not been previously characterized in histologic sections. Additionally, our work supports other groups that have shown astrocytes take up both tau monomers and oligomers [14, 17, 33]. We have built on this data by demonstrating isoform-specific uptake of tau monomers, which has, to our knowledge, not been previously described.

Our ability to comprehensively characterize astrocytic phenotypes in human tissue was limited by the relatively small number of channels in our RNAscope workflow. Because tufted astrocytes can have markedly reduced expression of GFAP (Togo & Dickson 2002), we also cannot exclude the possibility that our automated algorithm missed a small number of GFAP-negative tufted astrocytes, although we feel this is unlikely given our optimization strategy (see Methods). Although there was no global increase in astrocytic tau expression, the diffuse nature of astrocytic plaques compared to tufted astrocytes made it impossible for us to identify the soma of tau-positive astrocytes in CBD, and thus directly compare tau-positive and tau-negative astrocytes. Answering this question will require three-dimensional reconstruction in thicker sections or the application of tissue-clearing methods. Spatial transcriptomics combined with protein stains to identify tau-positive astrocytes also show great promise for in-depth astrocytic phenotyping in PSP and CBD. Our uptake assays are inherently limited due to their reliance on recombinant tau monomers, which do not necessarily recapitulate the post-translational modification patterns seen in the human brain. The relative toxicity of tau monomers and oligomers, and their respective contributions to astrocytic tau pathology, likewise remains unknown.

In summary, we have shown that astrocytes in PSP and CBD do not upregulate tau expression, that they preferentially take up 4R tau monomers in vitro, and that this process is impaired by nutritional and inflammatory stress. These findings strongly suggest that the increased levels of astrocytic tau seen in PSP and CBD are due to astrocytic uptake of neuronal tau rather than upregulation of astrocytic tau production and identify a potential mechanism for the observed isoform distribution of astrocytic tau aggregates. Future studies will be necessary to determine the molecular mechanisms for this uptake and its downstream effects on the recipient astrocytes.

## Methods

### Tissue procurement

Formalin-fixed paraffin-embedded (FFPE) tissue was obtained as previously described [11]. Individual cases used and demographic information are listed in Table 1. The University of Iowa’s Institutional Review Board determined that, since this project used tissue from deceased individuals exclusively, it does not represent human subjects research under the NIH common rule (determination #201706772). All methods were conducted in accordance with the relevant laws, regulations, guidelines, and ethical standards of our institution and with the 1964 Helsinki declaration and its later amendments or comparable ethical standards. Diagnoses were made according to the updated National Alzheimer’s Coordinating Center neuropathologic diagnostic criteria and other published guidelines [3, 5, 6, 15, 25]. In all cases we used frontal cortex as a readily available, standardized area expected to have tau pathology across all our diseases of interest.

**Table 1:** Case Information

Case ID	Pathologic Diagnosis	Age (yr)	Sex
#1	Control (SARS-CoV-19)	73	Female
#2	Control (cardiovascular disease)	67	Female
#3	Control (necrotizing fasciitis)	80	Female
#4	AD, high ADNC	58	Female
#5	AD, high ADNC	61	Female
#6	AD, high ADNC, LBD, and LATE	72	Male
#8	PSP	75	Male
#9	PSP	76	Male
#10	PSP	67	Female
#11	PSP	72	Female
#12	PSP	56	Male
#13	PSP	80	Male
#14	PSP	69	Female
#15	CBD	63	Female
#16	CBD	58	Male
#17	CBD	62	Male

*AD* Alzheimer’s disease; *ADNC* Alzheimer’s disease neuropathic change; *LBD* Lewy body dementia; *LATE* Limbic-predominant age-related TDP-43 encephalopathy; *PSP* Progressive supranuclear palsy; *CBD* Corticobasal degeneration

### Combined RNA in situ hybridization and immunofluorescence

RNA in situ hybridization (RNAscope) with immunofluorescence (IF) was performed on formalin-fixed, paraffin embedded (FFPE) tissue using the 3-plex Multiplex Fluorescent v2 Reagent Kit (Cat No. 323100, ACDBio) with the C1-*MAPT* probe (Cat. No 408991, ACDBio) according to the manufacturer’s protocol as previously described [11]. Briefly, 5 μm FFPE sections were baked for one hour at 75 °C, deparaffinized, and treated with hydrogen peroxide solution (manufacturer provided) for 10 min at room temperature. Antigen retrieval was done using a steamer for 15 min at 85°C, followed by 30 min of Protease Plus treatment at 40 °C in a HybEZ Oven (ACDBio; Newark, CA, USA). Probe hybridization and amplification were then done according to the manufacturer’s directions. We used the Cyanine 5 TSA fluorophore (NEL745E001KT; Perkin Elmer; Waltham, MA, USA) at 1:400. Slides were washed with TBST with 0.05% Tween20, blocked with normal goat serum and 0.1% bovine serum albumin (BSA) for 30 min at room temperature, then incubated with anti-GFAP (16825-1-AP, RRID: AB_2109646; Proteintech; Rosemont, IL, USA) and anti-phosphotau (AT8) antibodies diluted to 1:1000 in TBS + 0.1% BSA for 1.5 h at room temperature. This was followed by a 30-minute incubation at room temperature with goat anti-mouse IgG H&L Alexa Fluor 555 (ab150114, RRID: AB_2687594; Abcam; Cambridge, UK) and goat anti-rabbit IgG H&L Alexa Fluor 488 (ab150077, RRID: AB_2630356; Abcam; Cambridge, UK) antibodies, both at 1:1000 in TBS + 0.1% BSA. We used TrueBlack Lipofuscin Autofluorescence Quencher diluted according to the manufacturer’s guidelines (23007; Biotium; San Francisco, CA, USA) to quench autofluorescence, followed by 4′,6-diamidino-2-phenylindole (DAPI) and coverslipping with VectaShield Plus antifade mounting medium (H-1900; VectorLabs; Newark, CA, USA). We used ACDBio 3-plex positive and negative controls (Cat. no. 320861 and Cat. no. 320871, respectively; ACDBio; Newark, CA, USA), as well as an IF-only control to eliminate false-positive or false-negative results.

### Slide scanning and quantification

Slide scanning and quantification were done using a Cytation 5 from Agilent (Cat No. BTCYT5PW) with GEN5PRIME software (Cat No. BTGEN5IPRIM, Agilent) to create a customized protocol. Each set of experiments were run using identical protocol settings. A discovery scan was done to image the entire piece of tissue at 4X (Cat No. BT1320515, Agilent) using a combination of the DAPI filter cube (Cube: Cat No. BT1225100, Agilent; LED: Cat No. BT1225007, Agilent), GFP filter cube (Cube: Cat No. BT1225101, Agilent; LED: Cat No. BT1225001, Agilent), TRITC filter cube (Cube: Cat No. BT1225125, Agilent; LED: Cat No. BT1225012, Agilent), and CY5 filter cube (Cube: Cat No. BT1225105, Agilent; LED: Cat No. BT1225005, Agilent) as appropriate. Five regions of interest (ROIs) were selected at random in the cortex for each tissue using approximately equal sized ROIs, then each ROI was imaged in the appropriate channels using the above filters at 40X (Cat No. BT1320518, Agilent) and stitched using the GFP channel as a reference with 150 μm of overlap.

For quantification, subpopulations were defined in GEN5PRIME to identify cell types of interest and count the number of RNA puncta present. In our initial analysis comparing astrocytic tau expression between cases, astrocytes were defined as cells with a nucleus size of less than 15 based on DAPI and a mean GFP intensity of at least 10,000, regardless of the presence of tau pathology. Due to the diffuse nature of astrocytic plaques in CBD, it was not possible to definitively identify which astrocytic soma and nucleus corresponded to a set of tau-positive distal processes. For the comparison of tau positive and negative astrocytes within PSP cases, astrocytes with tau pathology were defined as cells that met the above criteria in addition to a mean TRITC intensity of at least 10,000, and astrocytes without tau pathology were defined as cells that met the above criteria with a mean TRITC intensity of less than 10,000. These settings were selected by a blinded observer so as to detect all morphologically identifiable tufted astrocytes in the AT8 channel (TRITC). RNA puncta were counted in each population based on the size of puncta (between 2 and 5 μm) using the CY5 channel. The average number of puncta in each subpopulation was calculated by GEN5PRIME using all five ROIs for each slide. P-values were assessed in GraphPad Prism using t-tests with each sample counting as one biological replicate, and graphs were made using R Studio and Adobe Illustrator. Data is presented as mean ± standard error of the mean (SEM).

### Stem cell procurement and culture

The H14 human embryonic stem cell (hESC) line was obtained from WiCell (Cat No. WAe014-A) and is listed as an NIH-approved line for research purposes (NIH Approval Number: NIHhESC-10-0064). Stem cell culture was done using a commercially available media kit (mTeSR Plus; Cat No. 100–0276, STEMCELL Technologies) following the manufacturer’s published protocol [29]. Briefly, cell colonies were grown on Matrigel-coated 6-well plates (Matrigel: Cat No. 354277, Corning; Plates: Cat No. CLS3516-50EA, Sigma Aldrich) in mTeSR Plus for at least three passages before being dissociated for downstream differentiation protocols. Matrigel was diluted according to the lot-specific dilution factor recommended by the manufacturer in 25 ml of DMEM/F12 HEPES (Cat No. 36254, STEMCELL Technologies) and set for 1 h at 37 °C before plates were used. Areas of cellular differentiation were removed each day from cultures. Passaging of colonies was done roughly every five days using Gentle Cell Dissociation Reagent (GCDR) from STEMCELL Technologies (Cat No. 100–0485). Stem cell pluripotency was validated using the STEMdiff Trilineage Differentiation Kit (STEMCELL Technologies, Cat No. 05230) and human stem cell antibody array from AbCam (Cat No. ab211066) according to the manufacturer’s directions.

### Generation of neural progenitor cells

Neural progenitor cells (NPCs) were created from the hESCs described above using the STEMdiff SMADi Neural Induction kit from STEMCELL Technologies (Cat No. 08581) according to their guidelines for Matrigel-coated 6-well plates [31]. Briefly, colonies were checked for areas of differentiation and removed, if necessary, before washing once with cold DPBS-/-. Colonies were then incubated for 8–10 min at 37 °C in GCDR for dissociation. Cells were collected and triturated to create a single-cell suspension, then spun down at 300 g for 5 min at room temperature. Cell counts were determined using a Countess 3 instrument (Cat No. AMQAX2000, Thermo Fisher), and 2,000,000 cells/well were plated in a 6-well plate in STEMdiff SMADi Neural Induction media. Daily media changes were performed for seven days followed by passaging of progenitor cells using Accutase (Cat No. 07920, STEMCELL Technologies), which was repeated for one additional passage (two passages total). Cells were plated at 1,500,000 cells/well in a 6-well plate for each passage. At passage 3, cells were either frozen down in CryoStor CS10 (Cat No. 07930, STEMCELL Technologies) at 3,000,000 cells/vial or moved onto the next step of the differentiation process.

### Generation of astrocyte precursor cells and mature astrocytes

Astrocyte precursor cells were created from the neural progenitor cells described above using the STEMdiff Astrocyte Differentiation media kit from STEMCELL Technologies (Cat No. 100 − 0013) following their protocol for Matrigel-coated 6-well plates [30]. Briefly, progenitor cells were plated at a seeding density of 1,900,000 cells/well in a Matrigel-coated 6-well plate and given daily media changes for seven days. For passaging, cells were incubated with Accutase for 8–10 min at 37 °C then spun down at 400 g for 5 min at room temperature. Cell counts were obtained using a Countess 3 instrument, and a density of 1,400,000 cells/well was used. Media changes were performed every other day for seven days, then the process was repeated once more (seeding density: 1,600,000 cells/well) for a total of two passages in the differentiation media. At passage 3, precursor cells were either frozen in CryoStor CS10 (above) at 2,000,000 cells/vial or moved onto the final stage of differentiation.

Mature astrocytes were created from astrocyte precursor cells using the STEMdiff Astrocyte Maturation media kit from STEMCELL Technologies (Cat No. 100 − 0016) following their guidelines for Matrigel-coated 6-well plates. Briefly, precursor cells were seeded at 1,900,000 cells/well in a 6-well plate, and media changes were performed every other day for seven days. Cells were dissociated with Accutase for 8–10 min at 37 °C before being spun down at 400 g for 5 min at room temperature. This process was repeated once more for 2 passages in the maturation media before cells were plated for downstream analysis.

### Immunocytochemistry

Primary and secondary antibodies used with their respective concentrations are shown in Table 2. Cells for ICC were grown in a 12-well plate (Cat No. 3513, Costar) on autoclave-sterilized 18 mm coverslips (Cat No. 12545100, Fisher Scientific) coated with Matrigel using the same protocol as described above. Coverslips were washed once with 1X PBS before fixing for 30 min with 10% formalin at room temperature and were subsequently washed twice with 1X PBS before storing in 1X PBS + 0.01% sodium azide at 4 °C. Coverslips were placed in a 12-well plate and incubated for 10 min at room temperature in extraction solution (0.02% Triton-X 100 in 1XPBS; Triton-X 100: Cat No. BP151, Fisher Scientific) before blocking for 30 min at room temperature in blocking solution (10% heat-inactivated fetal bovine serum (hiFBS) in 1XPBS; hiFBS: Cat No. S1245914, R&D Systems). Primary antibody incubation was done with the respective antibodies diluted in serum solution (1% hiFBS in 1XPBS) overnight at 4 °C with gentle rocking. Coverslips were washed five times for 1 min each at room temperature with 1X PBS, then incubated in secondary antibody diluted in serum solution for 1 h at room temperature protected from light with gentle rocking. Five 1-minute 1X PBS washes were performed before incubation with TrueView Autofluorescence Quenching solution (prepared according to manufacturer’s guidelines and diluted 1:10 with ddH_2_O) for 5 min with gentle agitation at room temperature protected from light. Coverslips were washed once more for 5 min with 1X PBS at room temperature with the addition of three drops of DAPI (Cat No. R37606, Invitrogen) then mounted on SuperFrost Plus slides (Cat No. 12-550-15, Fisher Scientific) using VECTASHIELD Plus mounting medium (Cat No. H-1900, Vector Laboratories).

**Table 2:** Antibody Product Information

Antibody	Source	Cat No.	RRID
AT8 (phospho-tau Ser202/Thr205)	Thermo Fisher	MN1020	RRID:AB_223647
GFAP	Proteintech	16825-1-AP	RRID:AB_2109646
NANOG	Cell Signaling Technology	4903T	RRID:AB_10559205
OCT3/4	Santa Cruz Biotechnology	sc-5279	RRID:AB_628051
PAX6	Biolegend	Poly19013	RRID:AB_2565003
SOX1	R&D Systems	AF3369	RRID:AB_2239879
Nestin	STEMCELL Technologies	#60091.1	RRID:AB_2905494
S100β	Sigma Aldrich	S2532	RRID:AB_477499
DCX	Cell Signaling Technology	4604 S	RRID:AB_561007
HT7 (total tau)	Thermo Fisher	MN1000	RRID:AB_2314654
Horse anti-mouse IgG Antibody Peroxidase	Vector Labs	PI-2000	RRID:AB_2336177
Goat anti-mouse IgG Alexa Fluor® 555	Abcam	ab150114	RRID:AB_2687594
Goat anti-rabbit IgG Alexa Fluor® 488	Abcam	ab150077	RRID:AB_2630356
Donkey anti-mouse IgG Cy3	Jackson Immunoresearch	715-165-150	RRID:AB_2313599
Donkey anti-rabbit IgG Alexa Fluor® 488	Jackson Immunoresearch	711-545-152	RRID:AB_2313584
Donkey anti-goat IgG Alexa Fluor® 647	Abcam	ab150135	RRID:AB_2687955

### Generation of plasmids

Custom human TauB and TauE plasmids were created on a pET29b backbone by GenScript on a fee-for-service basis, with 6xHIS and S tags added to the N- and C-terminal ends to ensure high purity of the resulting protein preparations. The resulting plasmids were validated by long-read sequencing (Plasmidsaurus), transformed into BL21 (DE3) OneShot Competent Cells (Cat No. C6000-03, Life Technologies), and given to the Carver College of Medicine’s Protein and Crystallography Facility at the University of Iowa for recombinant protein generation.

### Generation of recombinant tau

Recombinant human TauB (1N3R) and TauE (1N4R) proteins were produced at the Carver College of Medicine’s Protein and Crystallography Facility at the University of Iowa. Protein was produced in *E. coli* BL21 (DE3) cells (Cat No. 69450, Novagen) with the pET29b plasmids generated above. Bacteria were grown in Luria Broth (Cat No. 910-79-40-2, Research Products International) in the presence of kanamycin (100 ug/mL), and protein overexpression was induced with 1 mM IPTG in 3 h at 37 °C. Cells were disrupted by sonication and boiled at 75 °C for 15 min in buffer (50 mM NaPO_4_, 2 mM EDTA and 2 mM DTT, pH 6.8). All subsequent procedures were performed at 4 °C. Lysate was ultracentrifuged at 80,000 g for 45 min, and protein was purified by cation exchange chromatography (HiTrap SP, GE Healthcare), followed by size exclusion chromatography (HiLoad 16/600 Superdex 200 pg, GE Healthcare). Recombinant tau was labeled using an Alexa Fluor 647 Protein Labeling Kit (Cat No. A20173, Invitrogen) with 500 mg of recombinant protein input at a concentration of 1 mg/mL according to the manufacturer’s recommendations. Successful labeling was confirmed by measuring absorbance on a NanoDrop spectrophotometer.

### Western blotting

Western blots were run using 20ng of purified protein. Samples were run on a 10% Mini-PROTEAN TGX Stain-Free Precast gel (Cat No. 4561036, Bio-Rad) and blotted to PVDF. The membrane was blocked in EveryBlot blocking buffer (Cat No. 12010020, Bio-Rad) for 10 min at room temperature, then HT7 was diluted 1:5000 in EveryBlot and incubated with the membrane overnight at 4 °C (Table 2). Horseradish peroxidase-labeled horse anti-mouse secondary was used at 1:5000 diluted in EveryBlot for 1 h at room temperature and detected by Clarity Western ECL substrate (Cat. No 1705060, Bio-Rad) (Table 2). Chemiluminescence was measured using a ChemiDoc Touch Imaging System (Cat No. 1708371, Bio-Rad).

### Astrocyte plating for live cell imaging

Mature astrocytes were plated in glass bottom 12-well plates (Cat No. P12-1.5 H-N, Cellvis) at a density of 200,000 cells/well and allowed to settle for a minimum of 24 h before live-cell imaging. For experiments requiring different media conditions, cells were plated in either STEMDiff Astrocyte Maturation media without Supplement B (serum-starved) or STEMDiff Astrocyte Maturation media without Supplement B with the addition of “TIC” proinflammatory factors (30ng/mL TNF-alpha (Cat No. 300-01 A-10ug, Peprotech), 3ng/mL IL1-alpha (Cat No. 500-P21A-50ug, Peprotech), 400ng/mL C1q (Cat No. ab282858, Abcam) (TIC) [21]. Serum starvation occurred either at the beginning of the astrocyte maturation protocol (three weeks total) or for four days before live-cell imaging, and “TIC” induction occurred 24 h prior to live-cell imaging.

### Live cell imaging

Live-cell imaging was done using a Cytation 5 (above) with GEN5PRIME software (above) for 24 h using phase contrast with laser autofocus (Cat No. BT1225010, Agilent) and the CY5 filter cube (above) at 20X (Cat No. BT1320517, Agilent). Cells were incubated in the imager at 37 °C with 5% CO_2_ to mimic normal incubator conditions. A 5 × 5 matrix was used to capture 25 tiles across the center of the well with images captured every hour throughout the experiment. Images were processed in GEN5 to remove background fluorescence in the CY5 channel caused by phenol red in the media then tau puncta spots were counted by GEN5 using a minimum and maximum size cutoff (2.5 and 10 μm, respectively). Due to difficulties counting cells on phase contrast by GEN5, all phase contrast images for timepoint 1 (baseline) in each experiment were imported into Labscope (Zeiss). Cells were counted using the default settings of the AI Cell Counting Module (Zeiss), and counts were exported into a spreadsheet for data analysis.

### RNA sequencing

RNA extraction was performed from the mature hESC-derived astrocytes grown as a monolayer and used for live-cell imaging. Invitrogen’s PureLink RNA Mini kit (Cat No. 12183018 A) was used for RNA extraction according to the manufacturer’s protocol, and samples were sent to GeneWhiz for sequencing. For samples sent as cell pellets, cells were grown as described above, then scraped into cold DPBS-/- (above) using a cell spatula and spun down at 400 g for 5 min at 4 °C. The supernatant was discarded, and pellets were flash-frozen in a dry ice/ethanol slurry. RNA extraction and sequencing were done by GeneWhiz’s sequencing facility on a fee-for-service basis. Data analysis was conducted as previously described [28]. Briefly, RNA sequencing reads were aligned to the hg38 human reference genome using STAR aligner. Reads were quantified using the *subreads* package, and comparative gene expression analysis was done using the *DESeq2* package in R.

### Statistical analysis

Data analysis for RNA sequencing is described above. For other studies, data analysis was done by either a generalized linear model with a HAC correction for time dependence or using paired or unpaired t-tests, as appropriate. Total cell count, treatment group, and position in well were also included as independent variables for the generalized linear model. Generalized linear models were run in MATLAB, t-tests were done in GraphPad Prism, and graphs were generated using either R studio or GraphPad Prism. Network and gene ontology enrichment analysis was done using String-DB with standard settings.

### Figure creation

All figures were made using Adobe Illustrator or the BioRender imaging platform.

### Electronic supplementary material

Below is the link to the electronic supplementary material.

Supplementary Material 1

Supplementary Material 2: Table S1. List of all genes for Figure 4.

## Data Availability

All data and code generated over the course of this study are provided in the supplemental material.
