# Supplementary material for: Determinants of astrocytic pathology in stem cell models of primary tauopathies
Source: Acta Neuropathol Commun. 2023 Oct 6;11:161. doi: 10.1186/s40478-023-01655-1 (PMC10557325; doi:10.1186/s40478-023-01655-1)
Supplement: Supplementary file 1 — Supplementary Material 1 [file 40478_2023_1655_MOESM1_ESM.docx]

**Supplementary Material**

**Determinants of Astrocytic Pathology in Stem Cell Models of Primary Tauopathies**

Kimberly L. Fiock PhD^1,2^, Jordan Hook BS^1,4^, Marco M. Hefti MD*^1,2,3^

^1^Department of Pathology, University of Iowa, Iowa City, IA

^2^Experimental Pathology Graduate Program, University of Iowa, Iowa City, IA

^3^Iowa Neuroscience Institute, University of Iowa, Iowa City, IA

^4^Carver College of Medicine, University of Iowa, Iowa City, IA

*To whom correspondence should be addressed:

Marco M. Hefti, MD

25 S Grand Ave MRC-108-A

Iowa City, IA 52240

(319)-335-8427

[marco-hefti@uiowa.edu](mailto:marco-hefti@uiowa.edu)


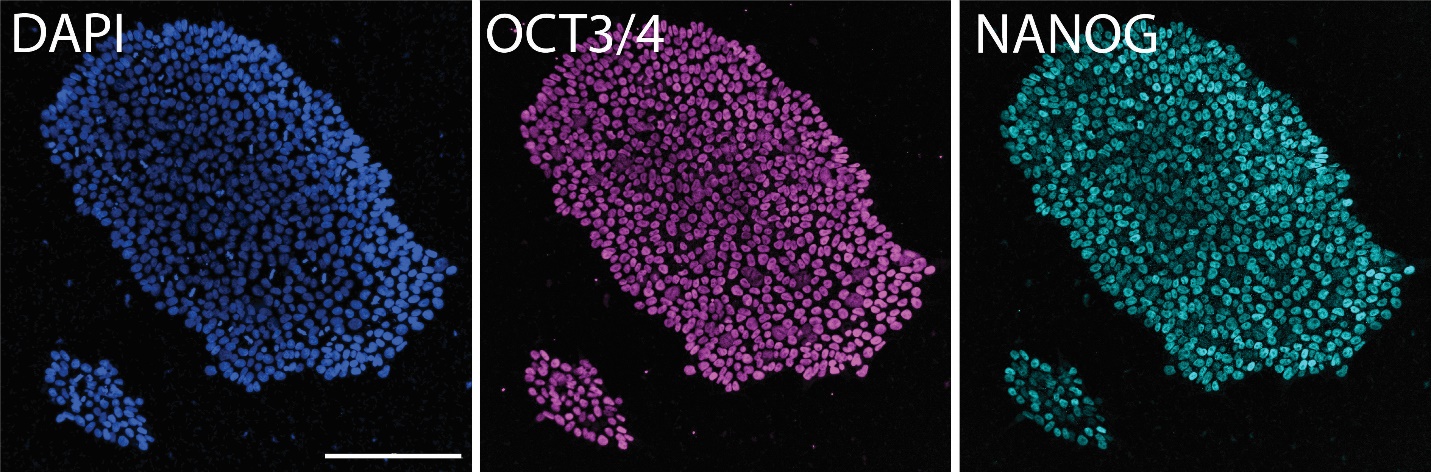


**Figure S1. Validation of stem cells by immunocytochemistry.** Channels shown separately for image in **Fig. 3**. Scale bar = 200 μm


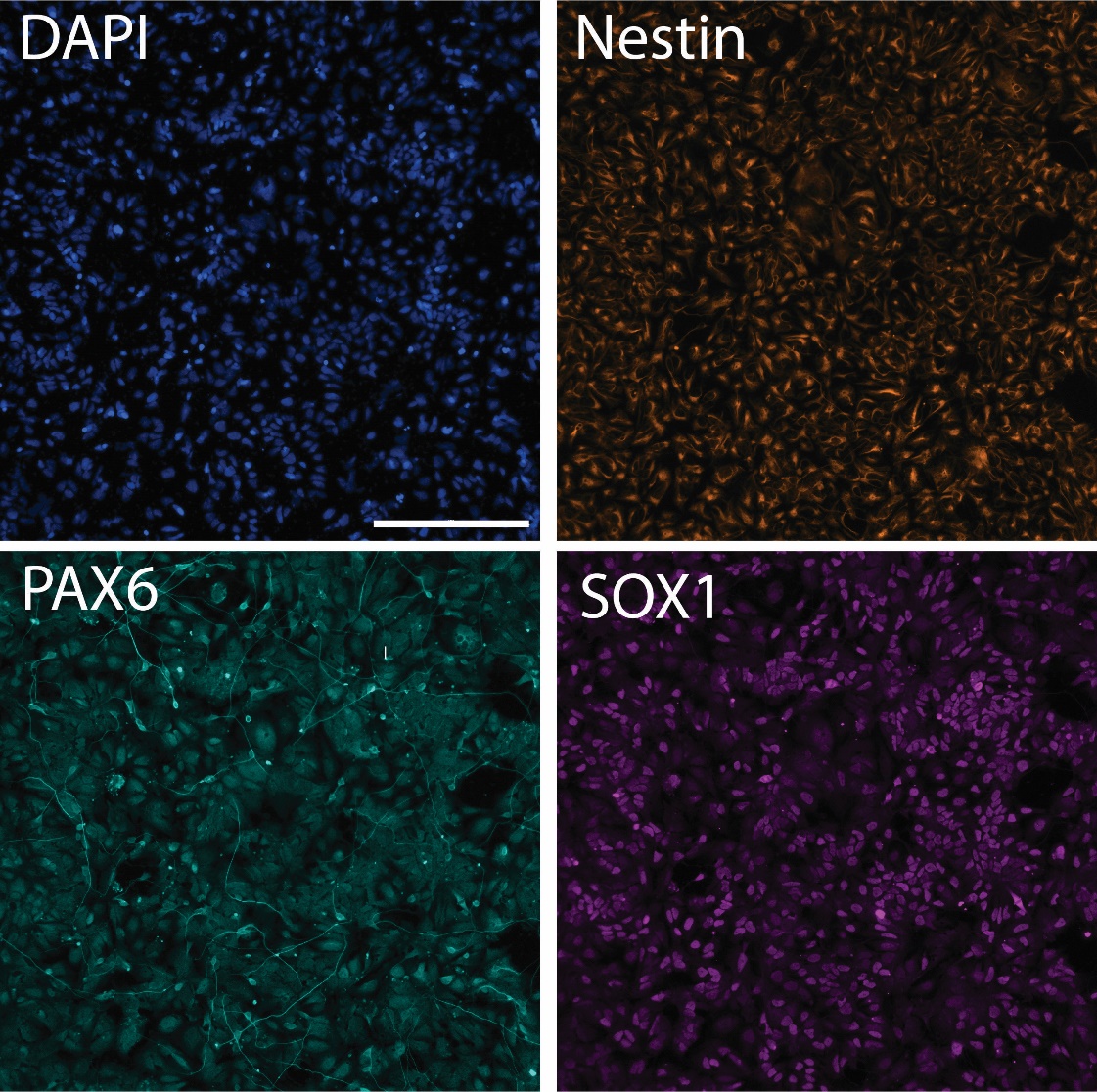


**Figure S2. Validation of neural progenitor cells by immunocytochemistry**. Channels shown separately for image in **Fig. 3**. Scale bar = 200 μm


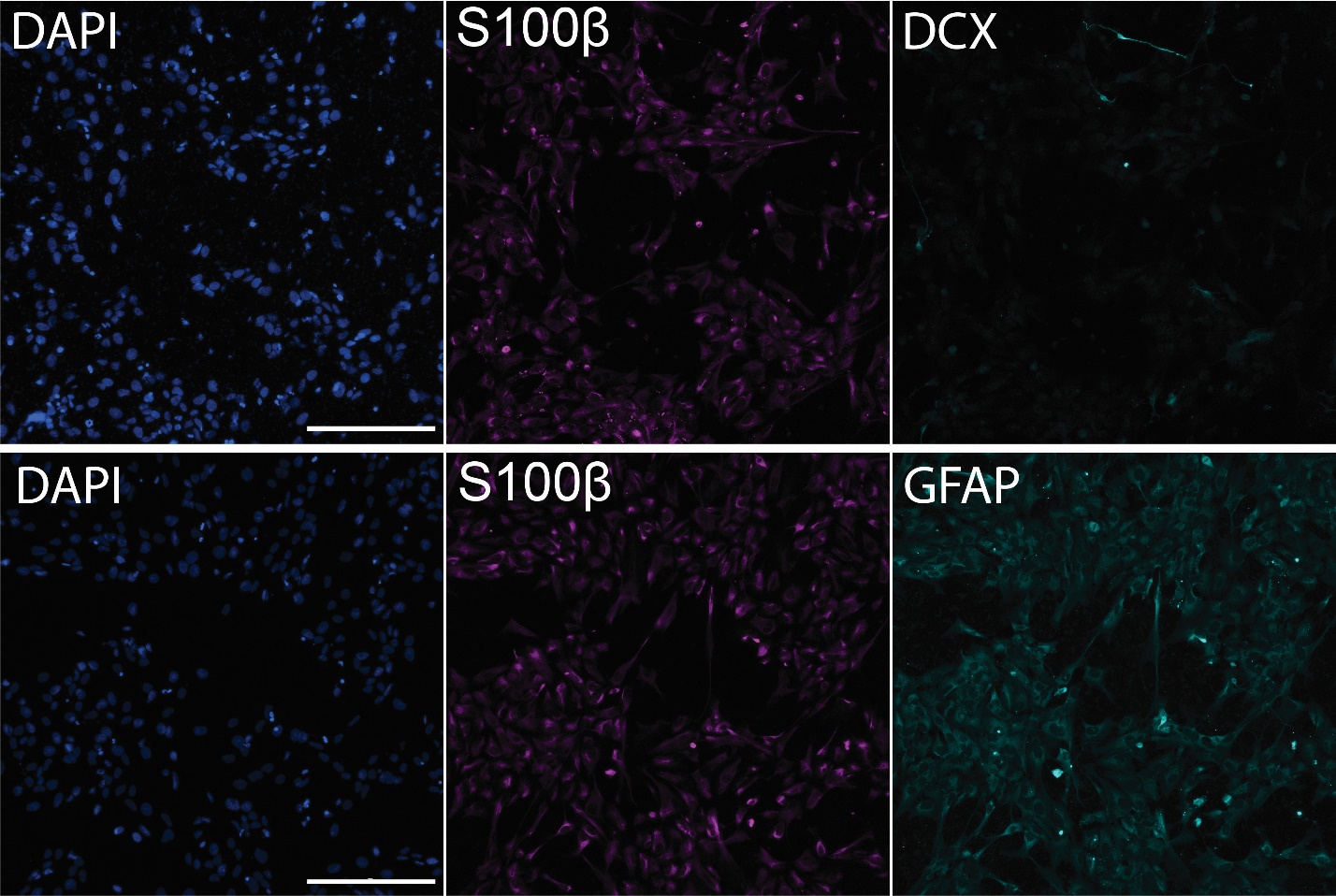


**Figure S3. Validation of astrocyte differentiation by immunocytochemistry.** Channels shown separately for image in **Fig. 3**. Scale bar = 200 μm

**
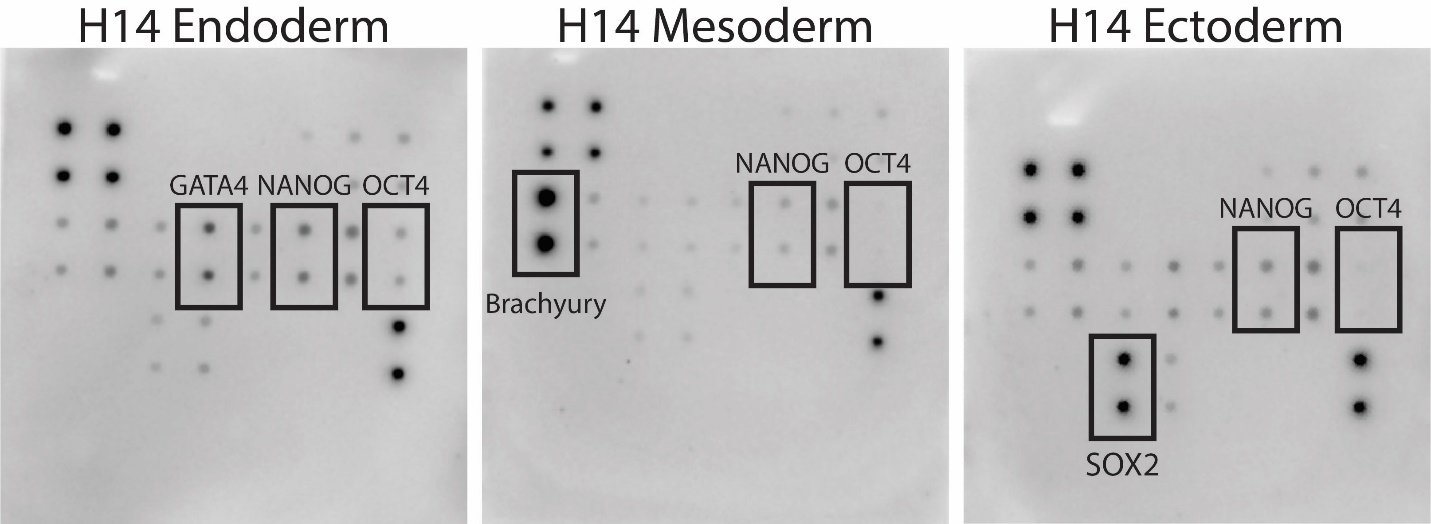
**

**Figure S4. Additional validation of stem cell pluripotency by trilineage differentiation.**

**
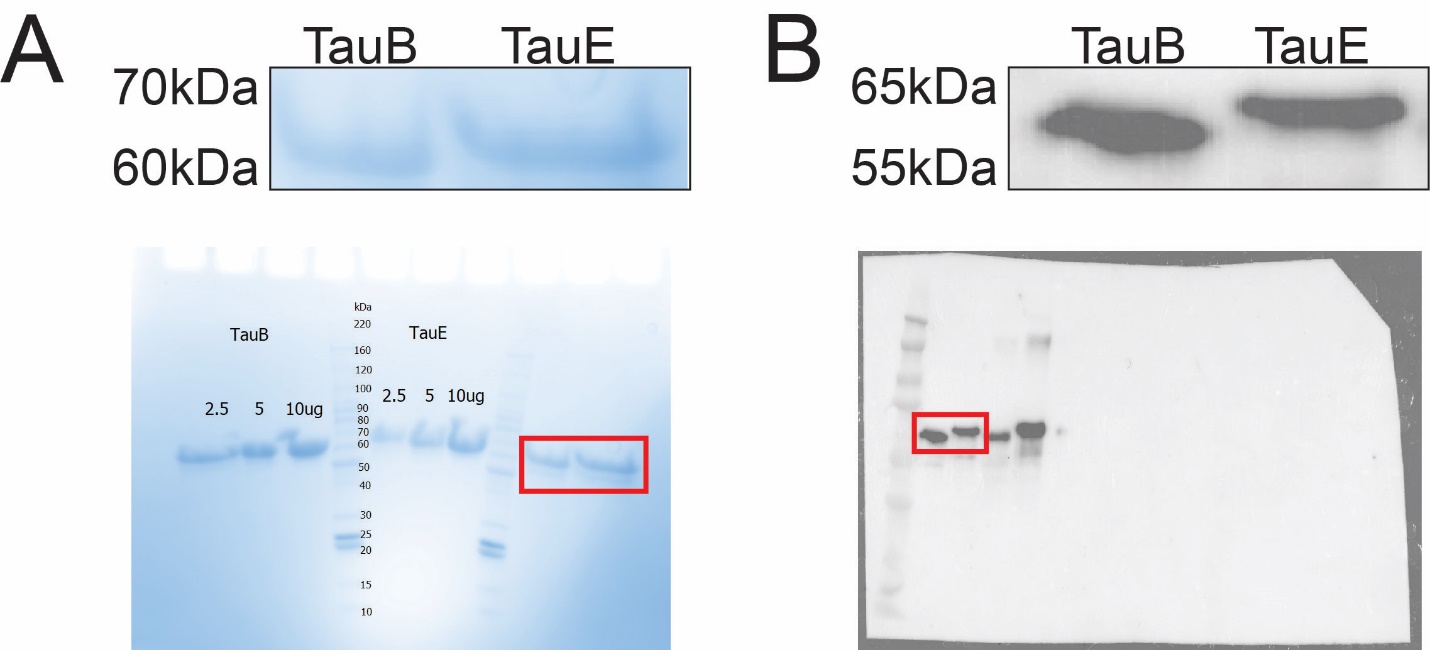
**

**Figure S5. Validation of recombinant tau.** (A) Recombinant TauE (1N4R) and TauB (1N3R) was run on an SDS-PAGE gel and stained with Coomassie Blue to assess for purity. Red box on whole gel represents magnified region shown above. (B) TauE and TauB were run on an SDS-PAGE gel, transferred to PVDF membrane and probed with anti-tau (HT7). Red box indicates relevant area magnified above

**
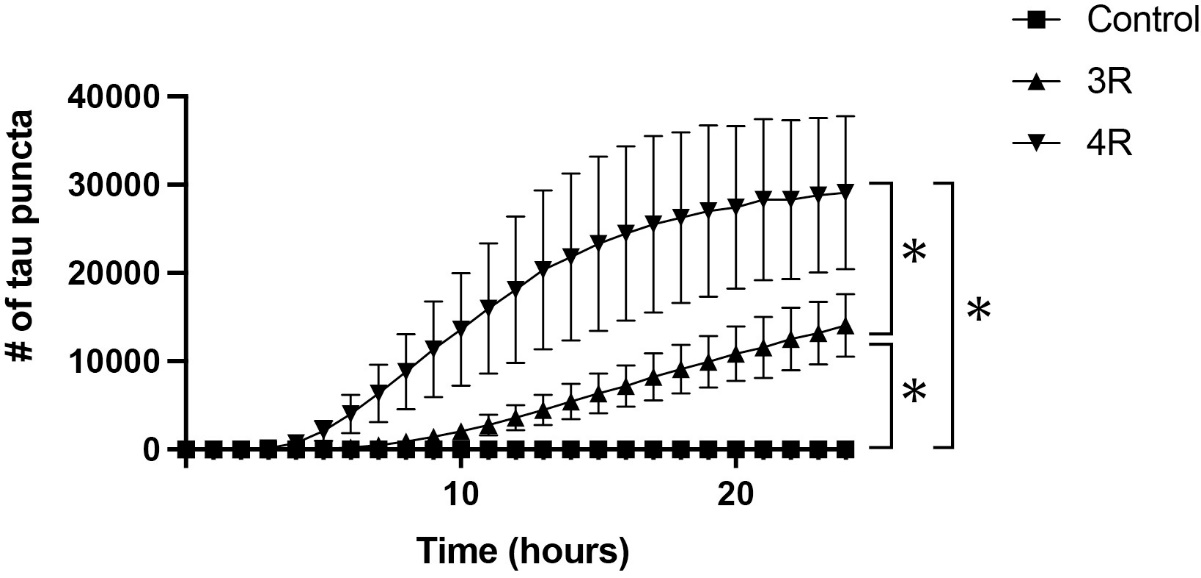
**

**Figure S6. Replication of Figure 3**. N=3 technical replicates per condition; *p<10-4
